# Access to healthcare insurance and healthcare services among syringe exchange program clients in Massachusetts: qualitative findings from health navigators with the iDU (“I do”) Care Collaborative

**DOI:** 10.1186/s12954-017-0151-4

**Published:** 2017-05-18

**Authors:** Thomas J. Stopka, Marguerite Hutcheson, Ashley Donahue

**Affiliations:** Department of Public Health and Community Medicine, Tufts University School of Medicine, 136 Harrison Avenue, Boston, MA 02111 USA

**Keywords:** Syringe exchange programs, Health insurance, People who inject drugs, Massachusetts, Substance use, Hepatitis C virus

## Abstract

**Background:**

Little is known about access to health insurance among people who inject drugs (PWID) who attend syringe exchange programs (SEPs). The goal of the current study was to assess perceptions of SEP staff, including health navigators and program managers, on access to health insurance and healthcare access among SEP clients following implementation of state and federal policies to enhance universal healthcare access in Massachusetts.

**Methods:**

Between December 2014 and January 2015, we conducted in-depth interviews (*n* = 14) with SEP staff, including both program managers and health navigators, to assess knowledge, attitudes, and beliefs related to health insurance enrollment and access to enhanced referrals among SEP clients. We developed a preliminary coding scheme from the interview guide and used a grounded theory approach to guide inclusion of subsequent thematic codes that emanated from the data. We analyzed the coded data thematically in an iterative fashion using a consensus-based approach.

**Results:**

We identified five primary themes that emerged from the qualitative interviews, including high levels of health insurance enrollment among SEP clients; barriers to enrolling in health insurance; highly needed referrals to services, including improved access to substance use disorder treatment and hepatitis C virus treatment; barriers to referring clients to these highly needed services; and recommendations for policy change.

**Conclusions:**

While barriers to enrollment and highly needed referrals remain, access to and enrollment in healthcare insurance plans among PWID at SEPs in Massachusetts are high. With the uncertain stability of the Affordable Care Act following the US presidential election of 2016, our findings summarize the opportunities and challenges that are connected to health insurance and healthcare access in Massachusetts. SEPs can play an important role in facilitating access to health insurance and enhancing access to preventive health and primary care.

## Background

Before the Affordable Care Act (ACA) was enacted, an estimated 22 million people aged 12 or older were classified as having alcohol or substance use disorders in the USA [1]. Approximately four of these 22 million (18%) individuals received some type of drug treatment [1]. The ACA’s goals of universal coverage and support of integrated primary care and drug treatment, including the requirement that substance use disorders be covered by health insurance, have helped many individuals suffering from substance use and misuse to get treatment [2–4]. The proportion of hospitalizations for substance use or mental health disorders in which the patient was uninsured, for instance, decreased from 22% in 2013 to approximately 14% in 2014, post implementation of the ACA, and decreases in uninsured hospitalizations for substance use and mental health disorders fell even further in states that expanded Medicaid under the ACA [5]. Still, healthcare utilization differs between insured and uninsured PWID and by injection frequency, and disparities by race/ethnicity exist [6]. While the ACA has increased healthcare coverage and access, additional steps and targeted interventions are needed to improve health outcomes among PWID to address disparities in coverage [6].

Healthcare reform was first enacted in Massachusetts (MA) in 2006, under Governor Mitt Romney. “Romney Care,” also called “Chapter 58: An Act Providing Access to Affordable, Quality, Accountable Health Care,” brought insurance coverage in MA up to 97% [7]. With passage of the federal ACA in 2010, also known as “ObamaCare,” universal healthcare continued in MA and became more achievable in many other states.

Studies of MA and other states that adopted universal healthcare prior to the ACA have shown that universal healthcare coverage did not necessarily translate into increased health insurance enrollment by people with substance use disorders [8]. In MA, there was no difference in utilization of substance use disorder (SUD) services before and after Chapter 58, which may have been due to a lack of expansion in the SUD treatment system [7]. Additionally, there were still barriers to enrolling in health insurance, including a lengthy enrollment process, citizenship documentation requirements, and patients losing health insurance due to changes in their contact information [7]. Even patients with health insurance experienced barriers to accessing drug treatment, including challenges affording copays [7]. While these challenges persisted in MA, in California, health insurance coverage was shown to be associated with shorter wait times to accessing treatment and longer treatment duration [9]. Since implementation of the ACA, coverage for SUD treatment has continued to vary across states, which could continue to contribute to inequities in access to covered care [10].

The USA is currently in the midst of an opioid epidemic, with substantial challenges tied to prescription opioid addiction [11], elevated heroin use [12], increasing hepatitis C virus (HCV) [13] and infectious endocarditis cases [14], especially in younger populations, and an overwhelming number of opioid overdose deaths [15, 16], and MA is no exception [17–19]. People who inject drugs (PWID), who are afflicted by these comorbidities at elevated rates, could benefit from enhanced access to health insurance that can facilitate access to treatment for SUD, HCV (with highly effective direct acting antiviral therapies), and other diseases. However, lack of affordability, stigma, long wait times to access treatment, limits on duration of treatment, lack of insurance coverage [20], and mental health comorbidities are still cited as barriers to treatment access and retention in treatment in communities across the USA, specifically among PWID [9, 20, 21].

Since the 1980s, syringe exchange programs (SEPs) have provided a wide range of services to PWID, acting as both harm reduction centers and facilitators to attaining culturally competent care [22]. Enhanced access to sterile syringes through SEPs has led to significant advances in the prevention of blood-borne disease transmission [23, 24]. SEPs have reduced injection-mediated risks (e.g., syringe sharing, syringe reuse) and have led to significant decreases in HIV incidence since they were first introduced in the 1980s [25, 26]. Over the years, SEPs have evolved to incorporate a wide range of wrap-around services, including access to preventive and clinical care, disease screening, referrals to SUD treatment programs, and entrée into healthcare services [27]. When SEPs have successfully linked PWID to treatment through culturally competent providers, overall healthcare utilization among PWID has been positively influenced by trust in physicians [9, 21, 28].

Many of the services SEPs provide, such as provision clean syringes and a number of harm reduction services, are typically available to clients regardless of whether or not they have health insurance. Other services and referrals, such as substance use disorder treatment and appointments with primary care doctors and specialists, may, without health insurance, be prohibitively expensive. Emergency department services can typically be accessed regardless of insurance status. Little is known about access to health insurance among PWID who attend SEPs. While SEPs have proven effective at increasing access to sterile syringes, decreasing HIV transmission and acquisition risk, and providing wrap around harm reduction and referrals services, there is a paucity of literature on access to insurance among SEP clients, which could enhance referrals to and treatment through clinical providers. The goal of the qualitative study we describe below was to assess knowledge, attitudes, and beliefs among health navigators and program managers at five authorized SEPs in MA related to health insurance access and enrollments, referrals to healthcare services for SEP clients, and experiences with the *iDU Care Collaborative* (described below). By assessing knowledge, attitudes, and beliefs among SEP staff, we attempted to close the current gap in literature on health insurance access among PWID.

## Methods

As part of the iDU Care (pronounced “I do care”) Collaborative, staff at five authorized SEPs, together with study staff at the Tufts University School of Medicine (TUSM), aimed to improve access to healthcare insurance among SEP clients through health insurance enrollments and re-enrollments, and enhanced referrals to medical (e.g., primary care providers) and social services. The project supported part-time staff members, referred to as “health navigators,” at each of the five authorized SEPs in MA operating at the time of the project. Health navigators assisted clients with enrollment and re-enrollment in publicly available health insurance (i.e., MassHealth, Medicaid in MA) and in accessing preventive care and treatment services.

Between December 2014 and January 2015, we conducted in-depth interviews (*n* = 14) with SEP staff who participated in the iDU Care Collaborative as either health navigators or program managers overseeing both iDU Care health navigators and SEP staff. Throughout this manuscript, we refer to program managers and health navigators collectively as “SEP staff” but differentiate quotes depending on whether the interviewee was a health navigator or a program manager. The five authorized SEPs operating at the time of the study and represented in the interviews included AIDS Action Committee (AAC) in Cambridge; Access, Harm Reduction, Overdose Prevention and Education (AHOPE) in Boston; AIDS Support Group of Cape Cod (ASGCC); and Tapestry Health in both Northampton and Holyoke. The interview guide included 17 open-ended questions focused on (1) experiences working with the iDU Care program to enroll PWID in health insurance, (2) healthcare services that could be provided to clients, (3) healthcare services that were difficult to provide to clients, (4) the healthcare referral process, (5) views on the impact of the ACA, (6) concerns about infectious diseases, and (7) recommendations for both public health policy and for the iDU Care program. Interviews were approximately 60 min long and were audio-recorded and subsequently transcribed using WavePedal7 software (Dictran, Purcellville, VA). Once interviews were initially transcribed, a second team member validated the transcription by re-listening to the digital recording while reading and making edits when necessary to ensure transcript accuracy.

We developed a preliminary coding scheme from the interview guide and used a grounded theory approach to guide inclusion of subsequent thematic codes that emanated from the data during coding of five initial in-depth interview transcripts. Three research assistants coded the five initial interviews and met regularly with the study PI to resolve substantive differences in coding of the qualitative data and to enhance inter-coder reliability. The coding scheme consisted of 15 parent codes and 74 child codes derived from themes that were consistently seen in interviews. Coding was conducted in NVivo10 (QSR International). After the completion of coding, we generated reports for major themes, which included challenges, health insurance enrollment and re-enrollment numbers, healthcare legislation, referrals, infectious disease, drug treatment, harm reduction, and recommendations. We analyzed the code-specific reports thematically in an iterative fashion using a consensus-based approach [29]. Upon completion of the 14 in-depth interviews, we reached saturation, whereby we detected no new additional themes and topics emanating from the data and concluded in-depth interviews. In this paper, we include presentation and discussion of relevant themes that were reported by at least three participants. Quotations from participants have been cleaned (e.g., removing terms such as “um,” “uh-huh,” “you know”; non-verbal cues; and pauses) for ease of reading. This study was approved by the Tufts University Health Sciences Institutional Review Board (IRB#: 11402).

## Results

We identified five primary and related themes that emerged from the qualitative interviews with syringe exchange staff working with the iDU Care Collaborative: high saturation of health insurance enrollment among SEP clients (97.3%), barriers to enrolling in health insurance, highly needed referrals to services, barriers to referring clients to these highly needed services, and recommendations for policy change.

### Saturation of Health Insurance Enrollment

Two primary goals of the iDU Care Collaborative were to enroll SEP clients in health insurance and to provide clients with health and social service referrals. While MA has had universal healthcare legislation since 2006, we anticipated that health insurance enrollment would be lower among PWID attending local SEPs. In 2014, when these interviews were conducted and several months after the iDU Care Collaborative had been in place, SEP staff from all sites reported that they found that almost all of their clients were already enrolled in health insurance before they were approached by iDU Care health navigators.

*The vast majority of our clients are enrolled and have primary care. It’s just very rare that it comes up that people need assistance in health insurance. Most people like I said… already have health insurance, whether it’s through the State [MassHealth] or not. I’d say about the entire time I’ve been here, I’ve probably helped 2 or 3 people sign up for health insurance.*

- Health Navigator, Cambridge

Staff reported that high saturation of health insurance among SEP clients was largely due to the fact that universal health insurance reform meant that clients had access to health insurance enrollment assistance through many other institutions and providers, such as homeless shelters, emergency rooms, prisons, and drop-in centers:

*Injection drug users, and our clients, have access to health care. They are being asked if they have health insurance at detox programs, at the needle exchange, in jail. Kind of everywhere they go they’re being asked if they have it, and if they don’t have it, then there is someone there that will step in and say, ‘Well, why don’t I help you.’*

- Health Navigator, Boston

### Barriers to enrolling in health insurance

For the small number of clients who did not have health insurance, SEP staff did report barriers to enrollment. Some barriers to enrollment cited by staff members focused on the health insurance enrollment system not working quickly or smoothly. Other barriers to enrollment were due to clients’ personal circumstances. Several staff reported that, for those clients who needed to enroll in health insurance, providing the required documentation was challenging:

*Because he had no job, he had no form of income, they would not give him health insurance. They told me that they weren’t gonna give health insurance to just anybody. [He] had to have proof of a Massachusetts residence, and sometime it’s hard with these clients…”*

- Health Navigator, Northampton

Staff also reported that many of those clients who attempted to enroll in health insurance were hampered by not having contact information. Clients could be homeless or experiencing housing instability and not have a mailing address, or they might not have a phone or email address. Not having these means of communication made it difficult for clients to stay in touch with the Health Connector, the health insurance enrollment system for MA, regarding any required follow-up communication and enrollment steps. SEP staff reported assisting clients in overcoming this barrier. For instance, staff reported helping clients set up email addresses, calling the Health Connector, letting clients use the SEP’s mailing address to receive forms, and assisting the client with follow-up:

*A lot of our folks are homeless, they’re not receiving mail anywhere. So it’s following up, and investing in that situation for the individual or helping them to make sure they are re-enrolling, and we have had successes in that area.*

- Program Manager, Cape Cod

*I am a little worried that you have to have an email address to fill out that form because I would say that probably the majority of our clients don’t have email, or if they have an email address they don’t have regular access to a computer. Some clients have a smart phone, but I feel like the chances of them holding on to that smart phone for long periods of time, you know, if you’re living in a shelter then theft does happen. Also if you’re actively getting high then forgetting your phone could happen, so I think relying on just internet applications would be unfortunate, especially with our population.*

- Health Navigator, Boston

Systemic issues with health insurance enrollment were also common barriers to staff being able to enroll clients in health insurance. These barriers included the time-consuming enrollment process, procedural changes due to the transition from MA Chapter 58 (i.e., RomneyCare) to the Federal ACA (i.e., ObamaCare), and the initial Health Connector website malfunction. Staff reported that it took at least 25 min and sometimes up to 2 h, to enroll clients in insurance by calling staff at the Health Connector.

*And then if you call, especially during the re-enrollment period, they don’t have enough staff. I’ve gotten reports, not just from my NEP [Needle Exchange Program] staff, but from the case managers on the HIV positive side that they’ve been on hold for couple hours, two hours, and when they finally get to someone they get hung up on. It’s really frustrating.*

- Program Manager, Cambridge

Enrolling clients through the Health Connector website was sometimes difficult, particularly because the website did not function well. For instance, there was no way to correct errors on the web-based forms required to apply for health insurance:

*We have a website that does not do the most basic functions that anyone with a small business can do on a website. And we spent a billion dollars for that. It blows my mind. It really does. You’re telling me that this, this, this is the best that we can do? A website that I can’t even go back and edit my profile afterwards? I can do that on ‘Joe Schmoe’ whatever website!*

- Program Manager, Cambridge

The steps in the process to enroll clients in health insurance changed as MA transitioned from Chapter 58 to the ACA. According to staff, the change in enrollment procedures between Chapter 58 and the ACA added an extra layer of difficulty in enrolling clients. The primary barrier was that staff had to re-learn how to enroll clients in health insurance under the different system. Additionally, under Chapter 58, some agencies had been able to enroll their clients in health insurance directly through a virtual network connecting their agency to the health insurance enrollment system. With the switch to the ACA, however, they could only assist clients in applying through the Health Connector.

*The other challenge was, until early January of 2014, we were able to enroll people directly into the Connector but with the changes that the State had to do to comply to the ACA we get bumped off and so we could no longer directly enroll people into health insurance. And I think since the changes in January, it’s taking a while to just kind of figure out what’s going on. You know that whole thing was just rolled out badly and it was really kind [of] challenging to know how to do it.*

- Program Manager, Northampton and Holyoke

### Highly needed referrals

SEP staff reported that they regularly referred clients to healthcare and social services. Some of these services were offered on-site at the SEP, while others involved outside providers and agencies. Staff reported that the most frequently needed referrals were SUD treatment, primary care, HCV treatment, mental health care, and housing assistance. They also mentioned that the number of referrals seemed to have increased substantially in recent years along with increases in SEP client numbers. Staff members often took a highly active role in providing referrals to clients.

*Referrals can be pretty far reaching. Again the most popular ones – or the most common ones would be treatment services, or detox services, mental health services to some extent, [and] housing.*

- Health Navigator, Cambridge

*The things that we refer to the most are primary treatment for STIs, substance abuse treatment, primary care, hep C treatment and shelters – those are the most common ones. Aside from the core things that we do - needle exchange, Narcan, testing - we’re a referrals center. We’re here to try to hook them up into other things, and bridge that gap. And that’s what we do, so we sit down with them, make the phone call for them, research it with them, give them ideas, we’ll drive them if possible, especially like we drive people to the detoxes*.

- Program Manager, Northampton and Holyoke

Drug treatment was the most often mentioned referral needed by clients. All SEP staff and program managers mentioned that they had experienced significant increases in the number of clients seeking SEP services during recent months and a particularly large and notably increased need for referrals to drug treatment:

*We’ve seen this huge increase in the amount of people who are asking for referrals to detox, methadone, suboxone, and vivitrol programs. It’s magnified ten times. There are so many people who understand that they do not have to continue to use, and we are going to help, we are going to deal with whatever the problems [are] right now, whatever the immediate need is, but if they are ready for treatment, or opiate replacement therapy, whatever, we are ready. And if you are not ready, let us know when you are and we can help you. So it’s [the iDU Care project] really been received really well by our clients as well.*

- Health Navigator, Cape Cod

HCV treatment was considered a highly needed referral, particularly in light of the new, highly effective direct acting antiviral HCV treatment options. Staff expressed a great deal of concern over the high rates of HCV among their clients.

*I would say a real high percentage, 70% maybe of people report having hep C. A lot less report being in treatment for it and care for it, than report having it. So there’s a big difference there. I think that’s the flavor of the day, if you will, because a lot of people have hep C, and a lot of people are not getting treated. And now, as we work with people that are trying to access treatment, they’re not just treating anybody that comes in the door.*

- Health Navigator, Boston

SEP staff and program managers also reported receiving many requests from clients for mental health services, primary care, and housing. They emphasized that access to these “basic services” were essential to provide clients with a stable base from which to address other pressing health challenges:

*Mental health is a big one… short term mental health intervention, and then also long term counseling and behavioral health stuff.*

- Program Manager, Boston

‘*I don’t have a primary, where do I go?’ So we do a lot of referrals for primaries, making appointments, picking a provider, helping people go get to their appointments by providing transportation, assistance with that.*

- Program Manager, Cambridge

*I’m going to say most needed [referrals are] treatments, certain medical [care], housing. Those are the most important. Until the person is able to have a more stable situation then they are able to address some of their other needs.*

- Program Manager, Cape Cod

### Barriers to referring clients

Staff noted both global and specific barriers towards successful referrals. Regardless of the type of referrals, staff expressed that finding providers who were culturally competent towards PWID was key. Interactions with culturally incompetent providers were a barrier towards continued client engagement in healthcare:

*But then it really depends on the quality of the, of the primary care that they get when they get there. So… if I need some medical stuff, and you give me a primary care doctor and I manage to get to the appointment… but then if I’m not well received… if there is. . . a bias…I guess against people that are, substance users…Then this really probably is not a good thing. And it begins to…take away from people’s participation. Rather than, than encourage*.

- Health Navigator, Boston

More specifically, two of the most needed referrals—SUD treatment and HCV treatment—were seen as two of the hardest referrals to achieve. For both SUD and HCV treatment, a low availability of providers and a related lack of timely services made it a challenge to successfully refer clients:

*It’s just really difficult to access the detox bed. Just none there. You know, so they tell people, ‘well come back tomorrow.’ But they’ve been here all day. So they, in the old days, there was a phrase ‘care on demand.’ It doesn’t really work like that right now.*

- Health Navigator, Boston

*If you’re mono-infected with hep C, you come on the list and unless you have pretty significant symptoms, you’re not really evaluated – until you get an intake six months later.*

- Program Manager, Northampton and Holyoke

Lack of health insurance or an inadequate health insurance plan with low coverage of SUD treatment was a barrier towards SUD treatment referrals:

*I would say, most important in terms of having insurance would be, referrals into treatment and detox.*

- Program Manager, Cape Cod

Insurance was also a barrier to accessing HCV treatment, primarily because insurance companies typically would not cover treatment except if the client’s disease had progressed to later stages of fibrosis (e.g., F3 or F4) or if the client had 6 months of sobriety or engagement in SUD treatment:

*Most insurance providers are requiring six months of abstinence, or treatment of some kind, methadone or suboxone. And we already talked about how many barriers there are just for getting onto treatment so then now you’re quadrupling the number of barriers; it’s just barriers upon barriers upon barriers.*

- Program Manager, Cambridge

### Policy and intervention recommendations

When asked about what policy changes or interventions staff would like to see, a common theme was reforming SUD treatment and insurance policies to provide adequate time for aftercare to enable clients to make a lasting recovery:

*But again, you know, 14 days, I think the conventional wisdom among most providers is that insurance needs to be covering treatments for an extended period of time. Minimum of 30 days. Minimum. And we don’t, we are not seeing that right now. And I think that that’s important if we want people to succeed in their recovery.*

- Program Manager, Cape Cod

Another salient recommendation was to increase access to HCV treatment for PWID:

*…like the hep C treatment. I’m really worried that even as the treatment becomes more accessible that the barriers will be put up that won’t allow people who are injecting to access it.*

- Program Director, Northampton and Holyoke

*These arbitrary six months on treatment – six months on drug treatment before you can access hep C treatment – it’s just ridiculous. I mean the fear among providers is risk of reinfection and treatments, and I get that they don’t want to invest if someone’s just going to re-infect. However, if somebody is connected to a needle exchange and they’re using clean works every time then there is no risk of reinfection. So that’s something that we need to have a conversation about, and we need to figure out how to make it cheaper.*

- Program Director, Cambridge

## Discussion

Universal healthcare in MA, starting with Chapter 58 (RomneyCare) in 2006 and continuing with the ACA (ObamaCare) in 2010, and the programs put in place to enroll people in health insurance through many different institutions, appear to have been highly effective in enrolling citizens from all walks of life in health insurance, including some of the most vulnerable populations.

We found that SEP staff across MA reported high levels of health insurance enrollment among their clients. Early expansion of universal healthcare appears to have paid large dividends, allowing enhanced access to health insurance broadly across the MA population. While SEP staff and program managers who we interviewed expressed concerns about some of the barriers and hurdles that needed to be surpassed to enroll new participants in health insurance, they acknowledged that the enrollment movement across a wide range of healthcare and social service agencies in MA provided multiple opportunities for PWID to be enrolled in health insurance in recent years. MA and other New England states reported substantial increases in SUD treatment following expansion of MassHealth (Medicaid) facilitated by Chapter 58 [30], and nationally, there is evidence that there have been continued opportunities for enhanced health insurance access through the ACA in a number of states across the USA. [31] While this is favorable news for MA, as well as a number of other states, not all states have had similar experiences, and challenges remain. In a recent study, researchers found that Medicare coverage of SUD treatment and treatment medications varied by state following ACA expansion of access to health insurance, and that disparities existed which could limit access to care among low income populations [10]. And, even with high access to health insurance among PWID in MA, SEP staff reported some of the same barriers to insurance enrollment among substance using populations that were identified in previous research, including documentation requirements, the lengthy enrollment process, and inadequate contact information [7]. Nevertheless, the enhanced access to health insurance coverage provides opportunities to people in the throes of addiction, including PWID, that have the potential to make substantial changes in health outcomes and remain as a legacy of Chapter 58 and the ACA [31].

While SEP staff reported good access to health insurance among PWID, this access did not always translate into good access to SUD and HCV treatment on the local level. SEP staff reported that having high-quality health insurance was a facilitator to accessing both SUD and HCV treatment, but larger barriers, such as low availability of SUD services, made it difficult to access these services, even with health insurance. This finding is in keeping with a previous study that suggested that healthcare reform in MA did not substantially increase access to SUD treatment, likely due to a low-capacity SUD treatment system [7]. In more recent years and subsequent to our interviews with SEP staff and program managers, however, access to improved SUD treatment tools and services have been reported in a number of states that have expanded access to health insurance and Medicaid [31].

SEP staff’s concern with low access to HCV treatment among PWID echoes findings from previous studies that demonstrated significant barriers to HCV treatment among PWID, including low rates of referral to an initial evaluation for HCV treatment, unwillingness of providers to treat active injectors, and delays in referring patients to specialists during the interferon era [32, 33]. With the advent of highly effective direct acting antiviral therapies, changes in insurance policies regarding eligibility to HCV treatment, and decreases in treatment costs as new competing direct-acting antivirals enter the market, there is increasing discussion about potential eradication of HCV [34–36]. While uptake of direct-acting antivirals among PWID has been limited by barriers at patient, provider, and systemic levels [37], there are signs of improvement. The American Association for the Study of Liver Diseases (AASLD) and the Infectious Diseases Society of America (IDSA), in their Recommendations for Testing, Managing, and Treating Hepatitis C indicate that:

“Treatment is recommended for all patients with chronic HCV infection, except those with short life expectancies that cannot be remediated by treating HCV, by transplantation, or by other directed therapy.” [38]

In their recommendation, AASLD and IDSA go on to state that

“…there are no data to support the utility of pretreatment screening for illicit drug or alcohol use in identifying a population more likely to successfully complete HCV therapy. These requirements should be abandoned, because they create barriers to treatment, add unnecessary cost and effort, and potentially exclude populations that are likely to obtain substantial benefit from therapy. Scale up of HCV treatment in persons who inject drugs is necessary to positively impact the HCV epidemic in the United States and globally.” [38]

In August of 2016, guidelines for MassHealth coverage changed, removing the sobriety and advanced liver damage requirements [39]. And clinicians have begun to report opportunities to treat PWID who have been on HCV treatment waiting lists for years.

Continuation of the ACA and its impact on hidden populations is uncertain following the US presidential election in November 2016. With threats of ending the ACA, it is difficult to surmise the future for health insurance and healthcare access in the USA for the general population, for people struggling with addiction, and for PWID. Considering the history of the ever evolving role of SEPs since the 1980s in the USA, we can be certain that SEP staff and program managers will continue to adapt to the legislative and public health landscape. SEPs will continue to play an important role in connecting clients to public health and clinical services and, depending on future legislation, may continue to be important players in the concerted effort to keep PWID insured, paving the way for access to needed healthcare services.

## Conclusions

State and federal policies to enhance access to healthcare insurance have enabled high levels of enrollment among PWID at SEPs in Massachusetts. Barriers to enrollment remain, and while the ACA has so far escaped the threat of repeal and replacement, its future stability remains uncertain following the presidential election of 2016. Our findings summarize the opportunities and challenges that are connected to health insurance and healthcare access in Massachusetts. SEPs can play an important role in facilitating access to health insurance and enhancing access to preventive health and primary care.
